# Seroprevalence of Merkel Cell Polyomavirus in the General Rural Population of Anyang, China

**DOI:** 10.1371/journal.pone.0106430

**Published:** 2014-09-03

**Authors:** Chanyuan Zhang, Fangfang Liu, Zhonghu He, Qiuju Deng, Yaqi Pan, Ying Liu, Chaoting Zhang, Tao Ning, Chuanhai Guo, Yongmei Liang, Ruiping Xu, Lixin Zhang, Hong Cai, Yang Ke

**Affiliations:** 1 Key Laboratory of Carcinogenesis and Translational Research (Ministry of Education), Laboratory of Genetics, Peking University Cancer Hospital and Institute, Beijing, People's Republic of China; 2 Anyang Cancer Hospital, Anyang, People's Republic of China; National Cancer Center, Japan

## Abstract

**Background:**

Despite the probably causal link between Merkel cell polyomavirus (MCPyV) infection and Merkel cell carcinoma (MCC), a rare but aggressive skin malignancy, little is known about the seroepidemiology of MCPyV among healthy adults in China.

**Methods:**

Serum antibodies against MCPyV were evaluated by multiplex serology in a population-based study of 5548 adults (including 1587 heterosexual couples) aged 25–65 years who were enrolled from rural Anyang, China in 2007–2009. Univariate and multivariate logistic regression analyses were performed to assess the risk factors for the seropositivity of MCPyV.

**Results:**

The seroprevalence for MCPyV was 61.0%. MCPyV seropositivity was significantly higher in males than in females (64.5% *vs.* 57.7%, *P*<0.001), and for both genders, showed a trend of increase with age (Male: *P*_trend_<0.001; Female: *P*_trend_<0.001). Furthermore, among antibody positives, antibody levels of MCPyV increased with advancing age (*P*_trend_ = 0.017). MCPyV seropositivity of one spouse was significantly associated with that of the other partner (Adjusted OR = 1.32, 95% CI: 1.07–1.62). However, there was no association between sexual behaviors and the seropositivity of MCPyV.

**Conclusions:**

High seroprevalence of MCPyV was observed in healthy Chinese individuals. Serological evidence suggests that nonsexual horizontal spread of MCPyV can occur among family members, and further research in this regard is needed.

## Introduction

Merkel cell polyomavirus (MCPyV), different from all the other human polyomaviruses so far, was classified as probably carcinogenic to humans, due to its unique relationship with Merkel cell carcinoma (MCC) [1]–[3]. MCC is a rare but aggressive skin malignancy arising in elderly or immunocompromised individuals [4], [5]. MCC cases not only occur among Caucasians as most frequently reported, patients of Asian ethnicity also appear in diverse regions of China and the possibility of under-diagnosis among Chinese cannot be excluded [6]. MCPyV DNA has been detected in approximately 80.0% of MCC tumors [1], [2], [7]. Involvement of MCPyV in the carcinogenesis of MCC is further supported by clonal integration of MCPyV within the tumor cell genome and the presence of mutations in large T antigen that prevents viral replication, but still sustains the transformational property [1], [8].

The carcinogenic potency renders MCPyV of considerable relevance for public health. Serologic studies indicate that MCPyV infection is highly prevalent (about 46.0%–88.0%) among healthy adults, which generally acquired asymptomatically in early childhood [9]–[16]. The variance in seroprevalence could be due to detection methods used, to differences in study populations, and to cut-off definitions. Only a few of large epidemiologic studies on MCPyV have been conducted mainly in western countries (the seroprevalence ranges from 46.0% to 80.0%) and data is especially limited in Asia [10], [13]–[16]. Except age and gender, few potential risk factors for MCPyV have been assessed among healthy populations. Till now, the exact transmission mode is not known, although MCPyV DNA has been found in low copy numbers in gastrointestinal, respiratory tract, and most frequently in cutaneous samples [14], [17]–[20].

This cross-sectional study aims to evaluate the prevalence and spousal concordance of MCPyV antibodies in a large rural Chinese population and to assess associated risk factors.

## Materials and Methods

### Study subjects

An ongoing population-based esophageal cancer cohort study in rural Anyang, China has been described elsewhere [21]. This MCPyV investigation was conducted in 6 of the 9 target villages cluster-sampled in the baseline of the cohort in 2007–2009. Eligibility criteria included: 25–65 years of age; permanent residents in one of the selected villages; no prior diagnosis of cancer, cardiovascular disease, immunocompromised disease, or mental disorder; and no history of infection with hepatitis B virus (HBV), hepatitis C virus (HCV), or human immunodeficiency virus (HIV). This study was approved by the Ethics Committee of School of Oncology, Peking University (Approval number: 2006020). Written informed consent was obtained from each participant who was included in the study, and the study protocol was followed according to the ethical guidelines of the 1975 Declaration of Helsink.

### Specimen and data collection

Five milliliters of peripheral blood were collected. After centrifugation, serum for antibody testing was temporarily stored at −20°C and later transported to Beijing, and stored at −80°C. A one-on-one computer-aided interview was administered by a trained interviewer. Information was collected regarding demographic characteristics, smoking (at least 1 cigarette per day for ≥ 12 months), drinking (drinking Chinese liquor at least twice per week for ≥ 12 months), personal hygiene habits, and sexual behaviors.

### Cloning of GST-VP1.FLAG fusion protein

The DNA construct of pGEX.MCPyV w156 was provided by Dr. Joseph J Carter (Fred Hutchinson Cancer Research Center). A FLAG tag was inserted into the pGEX-4T-1 plasmid, so that the major capsid protein (VP1) for MCPyV was expressed as fusion protein with N-terminal glutathione S-transferase (GST) and C-terminal FLAG tag. Primers listed in Table S1 were used to amplify the MCPyV VP1 sequence by KOD -Plus- Neo High fidelity PCR polymerase (Toyobo). PCR reaction conditions were carried out for 5 minutes at 98°C followed by 35 cycles of 98°C for 15 seconds, 58°C for 30 seconds, and 68°C for 90 seconds. The fragment was digested with *Eco*RI/*Xho*I, and then subcloned into the gel-purified pGEX-4T-1.FLAG plasmid. The recombinant clones were verified by sequencing (Sinogenomax Company, Beijing, China). Sequences were compared at the NCBI/BLAST web site (http://blast.ncbi.nlm.nih.gov/Blast.cgi).

### Expression of GST-VP1.FLAG fusion protein

GST-VP1.FLAG fusion protein was transformed into *E. coli* strain Rosetta (DE3) competent cell (Biomed Company). Fusion protein expression was induced at room temperature by 0.25 mM isopropyl-β-D-thiogalactopyranoside (IPTG) and bacteria were harvested after 12 hours incubation at room temperature. Clear lysate was prepared according to Sehr et al. [22], and was stored with 50% glycerol at −20°C. Fusion protein was characterized by Coomassie-stained SDS-PAGE and Western blot analyses using GST and FLAG tag-specific antibodies [23].

### Multiplex polyomavirus serology

This study adapted a multiplex serological assay based on GST fusion proteins, which was developed by Waterboer et al. for large-scale seroepidemiological studies [24]. Glutathione crosslinked to casein acted as a capture protein for GST, and was bound to fluorescence-labeled carboxylated magnetic beads (BIO-RAD). Each antigen was loaded onto specific bead sets with different colors. Serum specimens were diluted to 1∶50 and incubated with the bead mixtures overnight at 4°C followed by a 1-hour incubation at room temperature with shaking. Antibodies that bound to beads were detected with biotin-labeled anti-human IgG (H+L) (KPL, Gaithersburg, MD, USA) and streptavidin-R-phycoerythrin (Invitrogen). The bead mixtures were analyzed by the Bio-Plex 200 Instrument (BIO-RAD). Results were reported as median fluorescence intensity (MFI) of a minimum of 50 beads per bead set. Specific signals (net MFI) for MCPyV were calculated by subtracting the MFI for beads coated with GST alone.

GC beads binding of GST-VP1.FLAG fusion protein were quantified by an anti-FLAG M2 monoclonal antibody for each plate. Anti-FLAG tag MFI values among the testing days varied little (range 7351–14277 MFI for MCPyV). Within-day coefficients of variation (CVs) and between-day CV were 2.2%–13.3% (median, 7.5%) and 15.7%, respectively.

A cut-off value of 1000 MFI was set to determine the seropositivity for MCPyV. MFI values of MCPyV antibodies were defined to be high if they were in the 4th quartile among all the specimens tested. The high antibody level for MCPyV was MFI ≥ 15268.

### Statistical analysis

Potential risk factors that showed statistical significance in univariate logistic regression analyses, together with those reported exposure related variables were included in multivariate logistic regression models. Trend tests were conducted by treating ordered categorical variables as continuous covariates. All statistical analyses were performed using Stata for Windows (version 11.2, StataCorp, College Station, TX). The level of statistical significance was set at 0.05 (two-sided). All graphs were produced by the Prism program (GraphPad Software Inc, La Jolla, CA).

## Results

### Seroprevalence

**Figure 1:**
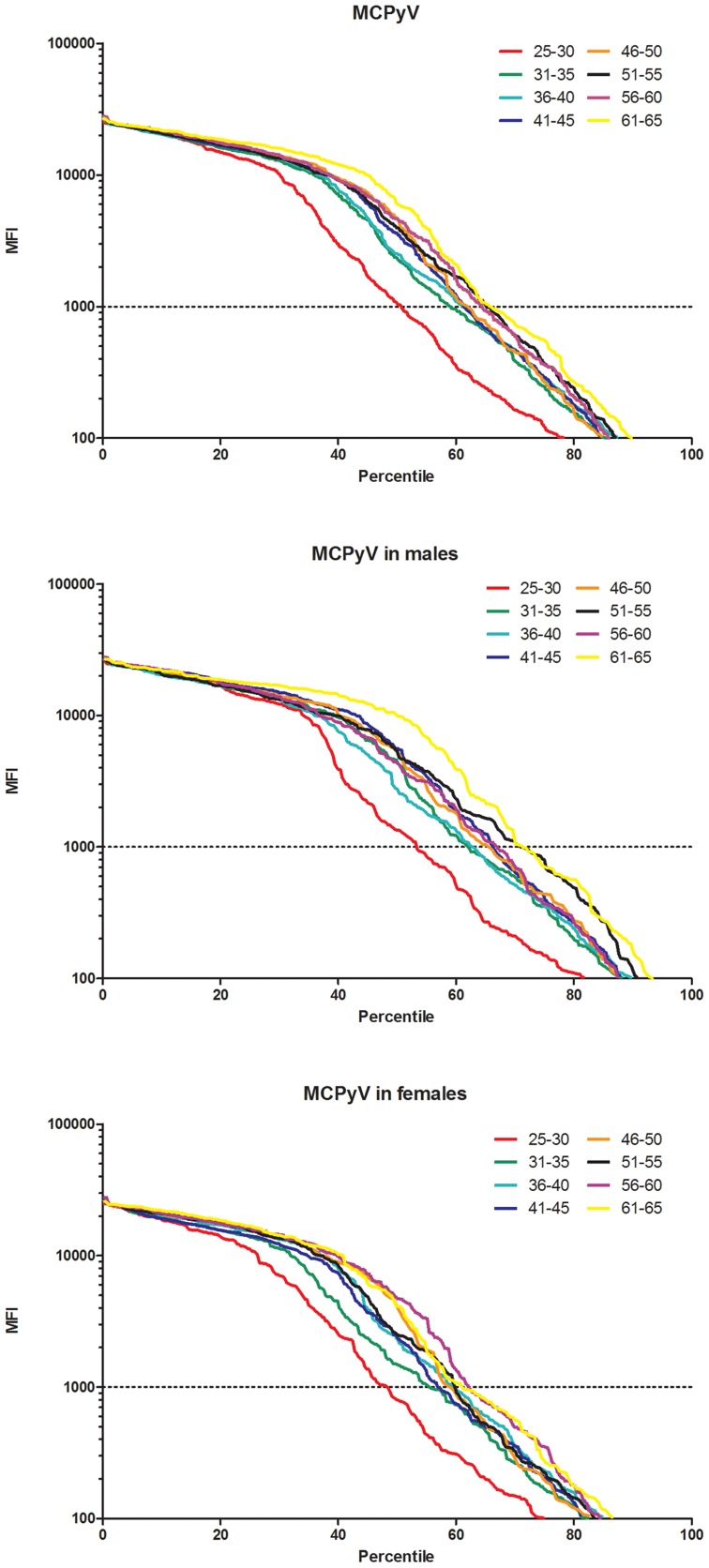
Distribution of the seroresponses for MCPyV by age and gender. Presented are seroresponses of 5548 healthy adults aged 25–65 years who were enrolled from rural Anyang, China, 2007–2009. The strength of the antibody reactions was plotted against the percentile according to age and gender. Color codes for age groups in years and the chosen cut-off of 1000 MFI are as indicated. The figure can be interpreted as follows (upper left panel, MCPyV antibodies among all subjects): For individuals aged 25–30 years, about 50% showed antibody reactions above 1000 MFI (that is, the seroprevalence of MCPyV is 50% using the cut-off of 1000 MFI) and about 30% above 10000 MFI (that is, the seroprevalence of MCPyV is 30% using the cut-off of 10000 MFI). **NOTE.** MCPyV: Merkel cell polyomavirus; MFI: mean fluorescence intensity.

Among 5548 participants, the overall seroprevalence for MCPyV was 61.0% (Table 1). The prevalence of antibodies to MCPyV was significantly higher in males than in females (64.5% *vs.* 57.7%, *P*<0.001), and for both genders, showed a trend of increase with age (Male: *P_trend_*<0.001; Female: *P_trend_*<0.001). These age-and-gender-dependent antibody reactivity patterns were independent of the cut-off values, according to Figure 1 (The strength of the antibody reactions was plotted against the percentile according to age and gender).

**Table 1:** Antibody positivity to MCPyV by age and gender in rural Anyang, China, 2007–2009.

Age (years)	% (No. of positive subjects/no. of subjects)		
	Total	Male	Female
25–30	50.8 (338/666)	53.3 (171/321)	48.4 (167/345)
31–35	59.0 (444/752)	62.2 (250/402)	55.4 (194/350)
36–40	61.2 (604/987)	62.9 (310/493)	59.5 (294/494)
41–45	61.4 (608/991)	66.3 (309/466)	57.0 (299/525)
46–50	62.0 (264/426)	65.5 (125/191)	59.2 (139/235)
51–55	65.2 (444/681)	71.0 (235/331)	59.7 (209/350)
56–60	64.4 (358/556)	66.9 (170/254)	62.3 (188/302)
61–65	65.9 (322/489)	71.3 (159/223)	61.3 (163/266)
Total	61.0 (3382/5548)	64.5 (1729/2681)	57.7 (1653/2867)
*P* value for trend^a^	<0.001	<0.001	<0.001

**NOTE.** MCPyV: Merkel cell polyomavirus.

a *P* values for trend were derived by logistic regression analyses considering categorical variables as continuous variables.

### Intensity of seroresponses

Although a majority of individuals in this population were seropositive for MCPyV, some adults displayed stronger antibody responses than others (Table 2, Figure S1). High antibody levels among MCPyV positive samples were positively associated with age, increasing from 38.1% for 25-to 35-year-old individuals to 45.0% for those aged 56 years and older (*P*_trend_ = 0.017) (Table 2).

**Table 2:** High levels of MCPyV in seropositive subjects according to age and gender in rural Anyang, China, 2007–2009.

Group	% (No. of subjects with high level/no. of positive subjects)	Crude OR (95% CI)^a^	*P* value^a^	Adjusted OR (95% CI)^b^	*P* value^b^
Age (years)					
25–35	38.1 (298/782)	1.00		1.00	
36–45	41.1 (498/1212)	1.13 (0.94–1.36)	0.184	1.13 (0.94–1.36)	0.182
46–55	40.3 (285/708)	1.10 (0.89–1.35)	0.397	1.10 (0.89–1.35)	0.392
56–65	45.0 (306/680)	1.33 (1.08–1.64)	0.008	1.33 (1.08–1.64)	0.007
*P* value for trend^c^		0.017		0.016	
Gender					
Male	41.3 (714/1729)	1.00		1.00	
Female	40.7 (673/1653)	0.98 (0.85–1.12)	0.731	0.97 (0.85–1.11)	0.661

**NOTE.** MCPyV: Merkel cell polyomavirus; OR: odds ratio; CI: confidence interval.

a Crude odds ratios, 95% confidence intervals and *P* values were derived by univariate logistic regression analyses.

b Adjusted odds ratios, 95% confidence intervals and *P* values were derived by multivariate logistic regression models including age and gender.

c *P* values for trend were derived by logistic regression analyses considering categorical variables as continuous variables.

### Risk factor analysis

The associations of MCPyV seropositivity with demographic and potential risk factors were shown in Table 3. Differences for MCPyV seropositivity were observed for types of employment, smoking, drinking, washing face before bed and bathing frequency in winter (Figure S2) in univariate analyses. However, after adjusting for age, gender and other potential confounders, only the effect of bathing frequency in winter remained. Individuals who bathed once every 15 days or more had a higher seropositivity of MCPyV than those who bathed at least once per week (Adjusted OR = 1.19; 95% CI: 1.01–1.39).

**Table 3:** Univariate and multivariate logistic analyses of risk factors for MCPyV seropositivity in rural Anyang, China, 2007–2009.

Variables	No. (%)	Crude OR (95% CI)^a^	Adjusted OR (95% CI)^b^
Age (years)			
25–35	782 (55.2)	1.00	1.00
36–45	1212 (61.3)	1.29 (1.12–1.48)	1.33 (1.15–1.55)
46–55	708 (64.0)	1.44 (1.23–1.70)	1.50 (1.24–1.82)
56–65	680 (65.1)	1.52 (1.29–1.79)	1.58 (1.27–1.95)
*P* value for trend^c^		<0.001	<0.001
Gender			
Male	1729 (64.5)	1.00	1.00
Female	1653 (57.7)	0.75 (0.67–0.84)	0.72 (0.60–0.87)
Education level			
Illiteracy, <1 y	456 (62.9)	1.00	1.00
Primary school, 1–6 y	1062 (61.9)	0.96 (0.80–1.15)	1.01 (0.83–1.23)
Junior high school, 7–9 y	1389 (59.1)	0.85 (0.72–1.01)	0.88 (0.72–1.09)
Senior high school or above, >9 y	273 (61.8)	0.95 (0.75–1.22)	0.93 (0.71–1.23)
*P* value for trend^c^		0.116	0.190
Marital status			
Being married or cohabiting	3010 (60.7)	1.00	1.00
Never married, divorced, separated or widowed	170 (62.7)	1.09 (0.85–1.41)	0.96 (0.74–1.26)
Types of employment			
Farming	1997 (59.5)	1.00	1.00
Non-farming	1183 (63.1)	1.16 (1.03–1.31)	1.15 (0.99–1.33)
Cigarette smoking			
Never	2178 (59.6)	1.00	1.00
Ever	1002 (63.5)	1.18 (1.05–1.34)	0.86 (0.72–1.03)
Alcohol consumption			
Never	2662 (60.2)	1.00	1.00
Ever	517 (64.2)	1.19 (1.02–1.39)	1.03 (0.86–1.23)
Smoke in the kitchen			
Smokeless	893 (61.1)	1.00	1.00
Smoky	1965 (60.5)	0.98 (0.86–1.11)	0.95 (0.83–1.08)
Very smoky	321 (61.4)	1.01 (0.82–1.24)	0.96 (0.77–1.18)
*P* value for trend^c^		0.944	0.500
Lifetime no. of sexual partners			
0–1	2873 (60.4)	1.00	1.00
2	138 (66.7)	1.31 (0.98–1.76)	1.28 (0.95–1.73)
≥3	136 (63.3)	1.13 (0.85–1.50)	1.09 (0.80–1.47)
*P* value for trend^c^		0.135	0.262
Age at sexual debut, years			
≤19	302 (61.4)	1.00	1.00
20–23	2153 (60.2)	0.95 (0.78–1.15)	0.99 (0.81–1.22)
≥24	715 (62.3)	1.04 (0.84–1.29)	1.00 (0.80–1.26)
*P* value for trend^c^		0.434	0.933
Frequency of sex per week			
None	904 (61.6)	1.00	1.00
1–2	1701 (60.7)	0.96 (0.85–1.10)	1.05 (0.90–1.22)
≥3	549 (59.2)	0.90 (0.76–1.07)	1.06 (0.87–1.29)
*P* value for trend^c^		0.249	0.581
Washing face before bed			
Never	2002 (62.3)	1.00	1.00
Occasionally	577 (58.2)	0.84 (0.73–0.98)	0.89 (0.77–1.04)
Frequently	593 (58.4)	0.85 (0.74–0.98)	1.00 (0.86–1.17)
*P* value for trend^c^		0.010	0.705
Interval between bathing in winter (days)			
1–7	579 (57.3)	1.00	1.00
8–14	812 (58.9)	1.07 (0.91–1.26)	1.09 (0.92–1.29)
≥15	1781 (63.0)	1.27 (1.09–1.46)	1.19 (1.01–1.39)
*P* value for trend^c^		0.001	0.035

**NOTE.** MCPyV: Merkel cell polyomavirus; OR: odds ratio; CI: confidence interval.

a Crude odds ratios and 95% confidence intervals were derived by univariate logistic regression analyses.

b Adjusted odds ratios and 95% confidence intervals were derived by multivariate logistic regression models including all the listed variables.

c *P* values for trend were derived by logistic regression analyses considering categorical variables as continuous variables.

### Concordance of heterosexual couples

Among 1587 heterosexual couples who both provided serum specimens, 607 (46.0%, 607/1320) had seropositive concordance for MCPyV (Table 4). MCPyV seropositivity of one spouse was significantly related to that of the other partner (Adjusted OR = 1.32; 95% CI: 1.07–1.62, *P* = 0.009).

**Table 4:** Spousal association of MCPyV seropositivity among 1587 heterosexual couples in rural Anyang, China, 2007–2009.

MCPyV serostatus of female partners	MCPyV serostatus of male partners		Total	Crude OR (95% CI)^a^	*P* value^a^	Adjusted OR (95% CI)^b^	*P* value^b^
	Negative	Positive					
Negative	267	424	691	1.00	0.008	1.00	0.009
Positive	289	607	896	1.32 (1.07–1.63)		1.32 (1.07–1.62)	

**NOTE.** MCPyV: Merkel cell polyomavirus; OR: odds ratio; CI: confidence interval.

a Crude odds ratios, 95% confidence intervals and *P* value were derived by univariate logistic regression analyses.

b Adjusted odds ratios, 95% confidence intervals and *P* value were derived by multivariate logistic regression models including the age difference between heterosexual couples.

## Discussion

In this population-based study, we determined the seroprevalence and associated risk factors for MCPyV in 5548 rural Chinese adults including 1587 heterosexual couples. Findings of this study increased our knowledge of the seroepidemiology and spousal correlation of this virus among the healthy population. To our best knowledge, this is the first report concerning MCPyV epidemiology from China.

This study showed that MCPyV circulated widely in the rural Chinese population, with a seroprevalence of 61.0%, which was in the middle seroprevalence range reported by previous studies from western countries (46.0%–88.0%) [9]–[16]. However, when comparing the prevalence data across studies, it is important to keep in mind that using different techniques may result in various prevalence figures. A variety of methods including enzyme-linked immunoassay (EIAs) and Luminex-based serological assays using virus-like particles (VLPs) or GST-VP1 recombinant protein have been employed to assess MCPyV seropositivity in previous studies [10], [12], [14], [25], [26]. Conformational epitope-based VLP EIAs are believed to be more sensitive than assays using VP1 recombinant protein [12], [27]. However, due to the ease of antigen production and purification as well as the comparable sensitivity, GST-VP1 based Luminex assay is an important tool for high-throughput analysis of human polyomaviruses antibodies in large-scale epidemiological studies [9], [16]. Nevertheless, as mentioned above, the possibility of lowering our estimate of MCPyV seroprevalence due to using GST-VP1 with less sensitivity still cannot be ruled out.

We observed higher MCPyV seroprevalence in men comparing to women, which implies that disparity in exposure or susceptibility to MCPyV with regard to gender may exist in this study population. MCPyV seroprevalence increased with advancing age ranging from 50.8% in the age group 25–30 years to 65.9% in the age group 61–65 years. This repeatedly reported age-dependence pattern indicates that MCPyV infection may be acquired throughout life or possibly be reactivated under conditions of waning immunity [14]. Furthermore, among antibody positives, a positive correlation between increased levels of MCPyV antibodies and age was observed. According to a longitudinal study, MCPyV antibody levels increased over time among two thirds of subjects who stayed seropositive up to 25 years after seroconversion [13]. The association between antibody levels and increasing age may reflect the fact that adults with high antibody levels had active viral replication, a state more commonly observed in the elderly due to waning immunity [15]. However, further research is deserved.

Population-based data regarding the seropositive determinants of MCPyV is lacking, although several case-control studies have examined the associations between MCPyV seroprevalence and potential risk factors in the control group [28], [29]. Except age, no other statistically significant associations have been consistently identified for MCPyV [13]. And till now, no definite transmission route for this virus has been demonstrated. The presence of MCPyV DNA has been most frequently detected in the normal skin of healthy populations, supporting the possibility of cutaneous transmission routes (shedding from the skin into the environment) [17], [18]. MCPyV may also be spread by a fecal-oral route or respiratory transmission, since MCPyV DNA can also be found in the aerodigestive tract and urban sewage [14], [19], [20]. Here we observed that, in line with other studies, antibody positivity against MCPyV was not related with sexual behaviors, supporting a nonsexual route of transmission [9]. Although the reliability of self-reports of sensitive sexual behaviors may be compromised by social desirability bias, significant association was found between sexual behaviors and male genital human papillomavirus (HPV) infection from this same cohort, in support of the validity of the survey questionnaires [30]. In addition, in one study which simultaneously measured both MCPyV and HPV antibodies, null association with the lifetime number of sexual partners was observed for MCPyV while strong association as expected for HPV was obtained [9]. Interestingly, we observed that infrequent bathing conferred an increased risk of MCPyV seropositivity, which has not been evaluated in previous studies. Worse personal hygiene (e.g. infrequent bathing) may increase the possibility of cutaneous transmission of MCPyV, and further study in this regard is needed.

Notably, this study fills a unique niche concerned with the spousal concordance for MCPyV seropositivity. Based on a large sample, our results showed that MCPyV seropositivity in one partner increased the risk of MCPyV seropositivity in the other. Frequent close contact (e.g. skin to skin) within couples and shared family environment is likely to increase the probability of nonsexual transmission of MCPyV via either respiratory, fecal-oral, or cutaneous routes. This may explain the presence of spousal correlation for MCPyV seropositivity. For deep exploration of MCPyV transmission modes, long-term longitudinal studies simultaneously detecting serum MCPyV antibodies and MCPyV DNA at multiple anatomical sites will be more informative.

MCPyV has gained the most attention, among all the polyomaviruses, due to its link to MCC. The MCPyV mutations found in MCC tumors kill the virus but nevertheless preserve its ability to transform cells [8], [31]. Additionally, MCPyV T antigen gene products, which target tumor suppressor proteins including retinoblastoma and p53, are specifically expressed in tumor cells [8], [32]. These indicate that MCPyV is not an incidental virus and partly explain how a common infection can result in a rare tumor. However, high seroprevalence of MCPyV as well as long incubation time between primary viral exposure (early in childhood) and occurrence of the tumor (a median age of approximately 70 years) suggest that additional events are required for MCPyV-related malignancy [2], [4], [10], [33]. Various hypotheses ranging from skin exposure to UV-light to perturbation of the immune system have been proposed, yet none have been elucidated [31]. Despite the limited understanding of the mechanism of MCPyV-induced carcinogenesis, the increasing incidence of MCC and poor prognosis of MCC patients warrants considering measures of targeting MCPyV to control MCC. Till now, several experimental vaccines have been developed to protect against MCPyV infection, which may consequently reduce MCC incidence [34]. Seroepidemiological studies provide important information concerning virus epidemiology and transmission and help to identify high-risk populations who may benefit from preventive measures such as vaccinations.

This study does have several limitations. Firstly, the cut-off used to define seropositivity in this study is arbitrary. Although Luminex-based serology reported signal magnitude of antibody reactions, quantitative interpretation of the signal strength was limited. According to Figure 1, the trend curves of signal intensity flattened off in the low percentiles, which may be due to that strongly seropositive sera began to saturate the beads at MFIs above about 7500. Using a single dilution of serum in the assay (such as 1∶50 used in this study) was likely to fail to satisfy the assumptions of the law of mass action for high titer individuals, which thus limited quantitative interpretation of high fluorescent signal intensity [11], [35]. Given this limitation, most of studies using Luminex-based serology focused on seropositivity determined by cut-offs instead of signal magnitude. However since there is no international standard serum, any cut-off definition is somewhat arbitrary and different results must be compared with caution. In this study, sensitivity analyses using different cut-offs showed that the findings were robust. Secondly, only antibodies against MCPyV w156 VP1 were measured, which may result in a lower estimate of MCPyV seroprevalence. Using VP1 proteins of representative local virus strains, which remain to be determined, may maximize the sensitivity of detection. Thirdly, information about potential risk factors for MCC such as skin sensitivity or markers of sun exposure were not collected, thus their association with MCPyV seropositivity could not be assessed. Finally, in order to clarify the modes of MCPyV transmission, analysis of concurrent data about serum MCPyV antibodies and MCPyV DNA at multiple anatomical sites both in adults and pediatric subjects would be more informative. Despite these limitations, this is the largest study characterizing serological profiles of MCPyV in general populations so far, and provides clues on relevant risk factors and transmission.

In summary, high prevalence of antibodies against MCPyV was observed in sera of healthy Chinese individuals. The seroprevalence and antibody levels of MCPyV increased with advancing age in adulthood. The absence of association between sexual behaviors and MCPyV seropositivity, along with the presence of spousal correlation of MCPyV seroprevalence, indicates the possibility of nonsexual horizontal spread of MCPyV between family members, and further research in this regard is needed.

## Supporting Information

Figure S1 **Intensity of the antibody responses for MCPyV by gender among seropositive individuals.** Scattergrams represent the distributions of the 3382 human sample reactivities with MCPyV (gray bars indicate medians) in rural Anyang, China, 2007-2009. Each dot corresponds to the MFI value of each serum sample. **NOTE.** MCPyV: Merkel cell polyomavirus; MFI: mean fluorescence intensity.
(TIF)

Figure S2 **Distribution of the seroresponses for MCPyV by bathing frequency in winter.** Presented are seroresponses of 5217 healthy adults (331 adults were excluded for missing information on bathing frequency) aged 25–65 years who were enrolled from rural Anyang, China, 2007–2009. The strength of the antibody reactions was plotted against the percentile according to bathing frequency in winter (interval between bathing in winter, days). Color codes for different groups (3 groups: bathed once every 1–7 days; bathed once every 8–14 days; and bathed once every 15 days or more) and the chosen cut-off of 1000 MFI are as indicated. **NOTE.** MCPyV: Merkel cell polyomavirus; MFI: mean fluorescence intensity.
(TIF)

Table S1 **Primers for amplifying MCPyV VP1 fragment.**
(DOC)
