# Supplementary figures and images for: Seroprevalence of Merkel Cell Polyomavirus in the General Rural Population of Anyang, China
Source: PLoS One. 2014 Sep 3;9(9):e106430. doi: 10.1371/journal.pone.0106430 (PMC4153645; doi:10.1371/journal.pone.0106430)

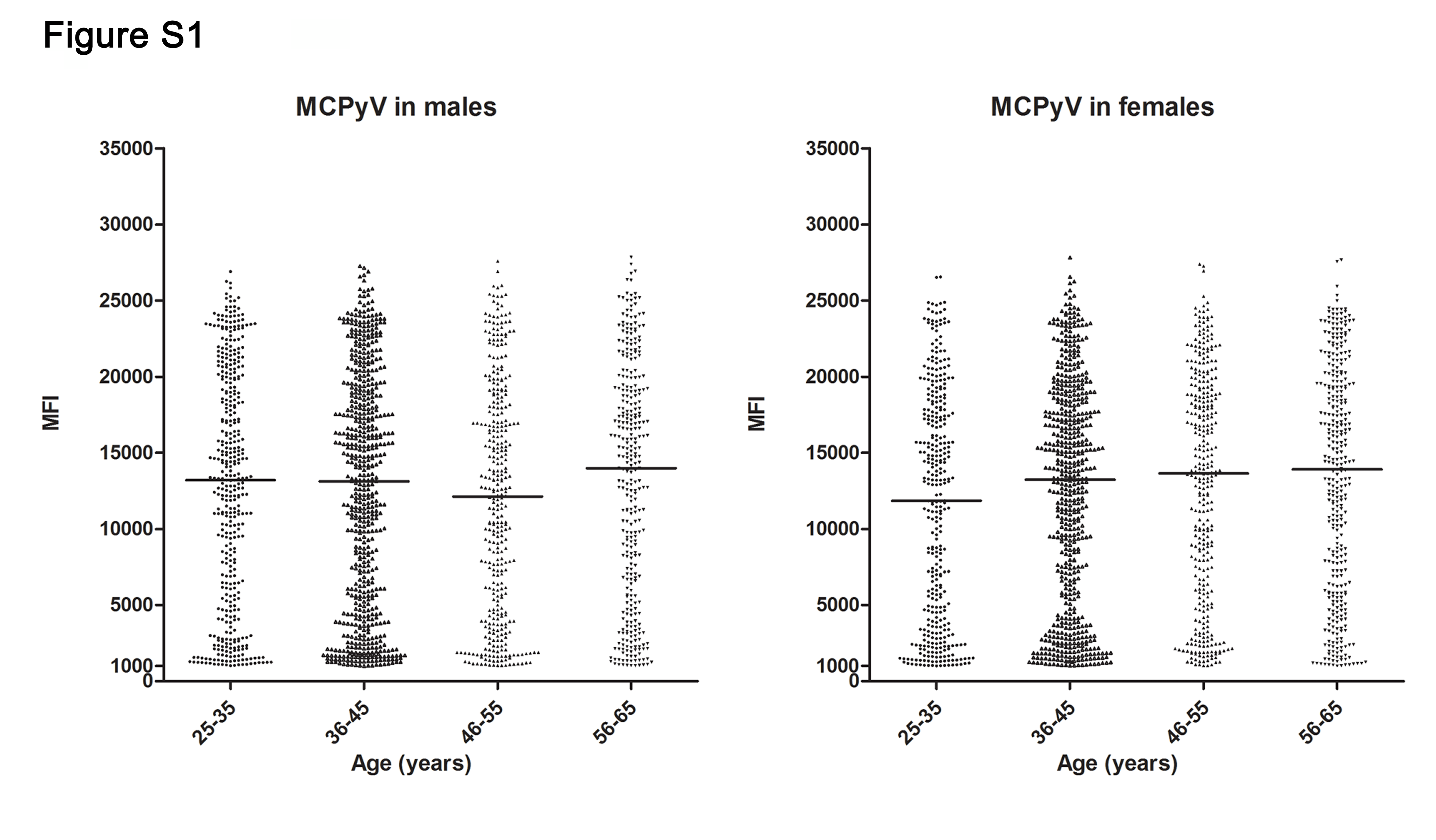

Supplement: Figure S1 — Intensity of the antibody responses for MCPyV by gender among seropositive individuals. Scattergrams represent the distributions of the 3382 human sample reactivities with MCPyV (gray bars indicate medians) in rural Anyang, China, 2007-2009. Each dot corresponds to the MFI value of each serum sample. NOTE. MCPyV: Merkel cell polyomavirus; MFI: mean fluorescence intensity. (TIF) [file pone.0106430.s001.tif]

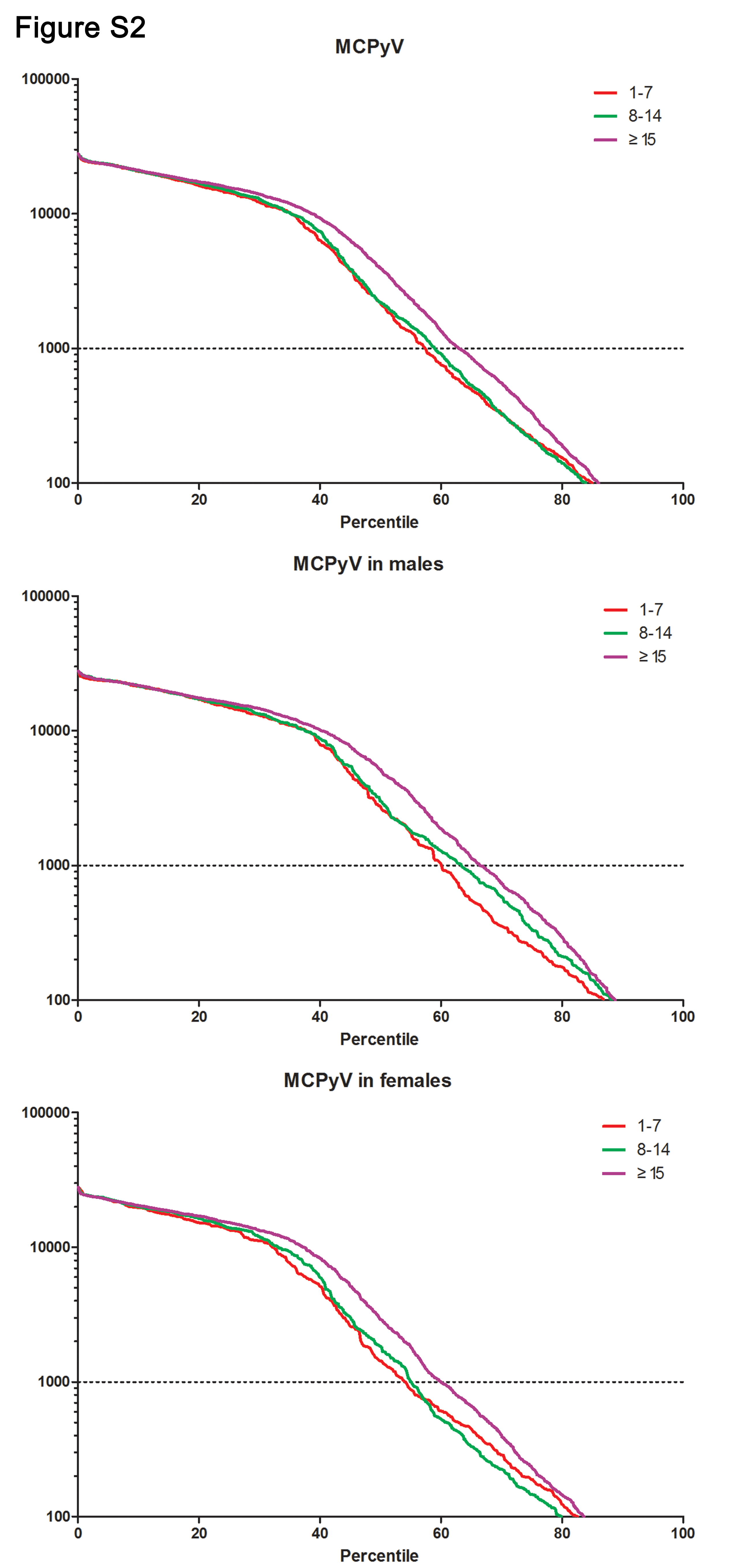

Supplement: Figure S2 — Distribution of the seroresponses for MCPyV by bathing frequency in winter. Presented are seroresponses of 5217 healthy adults (331 adults were excluded for missing information on bathing frequency) aged 25–65 years who were enrolled from rural Anyang, China, 2007–2009. The strength of the antibody reactions was plotted against the percentile according to bathing frequency in winter (interval between bathing in winter, days). Color codes for different groups (3 groups: bathed once every 1–7 days; bathed once every 8–14 days; and bathed once every 15 days or more) and the chosen cut-off of 1000 MFI are as indicated. NOTE. MCPyV: Merkel cell polyomavirus; MFI: mean fluorescence intensity. (TIF) [file pone.0106430.s002.tif]
