# Supplementary material for: Seroprevalence of Merkel Cell Polyomavirus in the General Rural Population of Anyang, China
Source: PLoS One. 2014 Sep 3;9(9):e106430. doi: 10.1371/journal.pone.0106430 (PMC4153645; doi:10.1371/journal.pone.0106430)
Supplement: Table S1 — Primers for amplifying MCPyV VP1 fragment. (DOC) [file pone.0106430.s003.doc]

| **Table S1. Primers for amplifying MCPyV VP1 fragment** | | | |
| --- | --- | --- | --- |
| Direction | Primer sequence 5'-3'^a^ | Restriction sites | Product length (bp) |
| Forward | CCGGAATTCGCACCAAAAAGAAAAGCATCATCC | *Eco*RI | 1272 |
| Reverse | CCGCTCGAGTAATTCTTGTGTTTGGCTTTCTTTTTGAG | *Xho*I |
| **NOTE.** MCPyV: Merkel cell polyomavirus; bp: base pair. | | | |
| ^a^ Sequences underlined are the restriction sites. | | | |
